# Perspectives of ERCC1 in early-stage and advanced cervical cancer: From experiments to clinical applications

**DOI:** 10.3389/fimmu.2022.1065379

**Published:** 2023-01-11

**Authors:** Pei Du, Guangqing Li, Lu Wu, Minger Huang

**Affiliations:** Department of Gynaecology and Obstetrics, Guangzhou Panyu Central Hospital, Guangzhou, Guangdong, China

**Keywords:** ercc1, cervical cancer, cisplatin, survival, mechanism

## Abstract

Cervical cancer is a public health problem of extensive clinical importance. Excision repair cross-complementation group 1 (ERCC1) was found to be a promising biomarker of cervical cancer over the years. At present, there is no relevant review article that summarizes such evidence. In this review, nineteen eligible studies were included for evaluation and data extraction. Based on the data from clinical and experimental studies, ERCC1 plays a key role in the progression of carcinoma of the uterine cervix and the therapeutic response of chemoradiotherapy. The majority of the included studies (13/19, 68%) suggested that ERCC1 played a pro-oncogenic role in both early-stage and advanced cervical cancer. High expression of ERCC1 was found to be associated with the poor survival rates of the patients. ERCC1 polymorphism analyses demonstrated that ERCC1 might be a useful tool for predicting the risk of cervical cancer and the treatment-related toxicities. Experimental studies indicated that the biological effects exerted by ERCC1 in cervical cancer might be mediated by its associated genes and affected signaling pathways (i.e., XPF, TUBB3, and. To move towards clinical applications by targeting ERCC1 in cervical cancer, more clinical, *in-vitro*, and *in-vivo* investigations are still warranted in the future.

## Introduction

Despite an upward trend in the HPV vaccination rates, cervical cancer remains the fourth most common female cancer worldwide (1, 2). Cervical cancer accounts for 527,600 new cases, representing 5% of all new cancer cases, and around 265,700 deaths annually worldwide (3). Patients with cervical cancer tend to metastasize early, resulting in a poor prognosis and a low 5-year survival rate of 30-60% (4). The major cause of it is infection with High-risk Human Papillomavirus and its diagnosis requires histopathological evaluation. Radical hysterectomy remains the first-choice therapy for patients at an early stage. A growing number of young patients have been diagnosed with this disease in recent years (5). As a result, some patients wish to preserve their fertility. In the late 1980s, the radical vaginal trachelectomy with bilateral pelvic lymphadenectomy was proposed as one of the standard approaches for fertility-sparing treatment (6). As for locally advanced cervical cancer, platinum-based concurrent chemoradiotherapy remain the gold-standard of treatment (7).It is problematic to treat locally advanced cervical cancers at stage IIb of the Federation of Gynecology and Obstetrics (FIGO). It invades the parametrium and lymph node, and is usually considered inoperable. Several studies demonstrated that neoadjuvant chemotherapy (NAC) reduced the tumor volume and increased tumor resectability, which achieved satisfactory outcomes in locally advanced cervical cancer (8, 9). As known, resistance to chemotherapy is the main obstacle to locally advanced cervical cancer treatment (10). Therefore, it is urgent to identify the biomarkers to predict chemotherapy or NAC response in locally advanced cervical cancer.

Excision repair cross-complementation group 1 (ERCC1) (the DNA repair gene) is a gene associated with platinum sensitivity and has been proposed as a novel biomarker of cervical cancer over the years (11, 12). ERCC1 gene is located on 19q13.2-q13.3, and encodes a 297 amino acid protein (13, 14). The C-terminal domain of ERCC1 interacts with xeroderma pigmentosum group F (XPF), which forms a heterodimeric protein complex. The complex is considered to be the main component of the nucleotide excision repair (NER) pathway (15). There are several major pathways for repairing DNA damage in human cells, one of which is NER (16). It can remove great varieties of helix-distorting DNA lesions, including UV-induced pyrimidine dimers, bulky chemical adducts, and photoproducts (17). The NER complex stabilizes the unwound DNA intermediate by recruiting xeroderma pigmentosum group A and replication protein A (18). Cisplatin is an alkylating compound that exerts its cytotoxic action by interfering with DNA replication by forming strong intrastructural cross-links, which activates cell apoptosis (19). Therefore, ERCC1 overexpression may have an adverse impact on cisplatin-induced cell death. Conversely, the inhibition of ERCC1 may sensitize cancer cells to cisplatin. In a study reported by Kassem et al. (20) on 80 colorectal cancer patients who received first-line oxaliplatin-based chemotherapy, patients with low ERCC1 expression had longer overall survival than those with high ERCC1 expression (P=0.011). Similarly, Torii et al. (21) also demonstrated that the expression level of ERCC1 was significantly increased by cisplatin treatment. They also found an association between ERCC1 expression and chemotherapeutic sensitivity of cervical adenocarcinoma cells. Additionally, a case-control study showed that low expression of ERCC1 was closely related to a significantly increased risk for cervical cancer (22). Though ERCC1 can be used not only as a prognostic biomarker but also to identify patients who will benefit from chemotherapy, the evidence has been debatable (23). In this present study, we summarize all published clinical and experimental data on ERCC1 applications in cervical cancer.

## ERCC1 in cervical cancer

### Roles of ERCC1 in cervical cancer among the current relevant studies

A systematic search was conducted in four databases, including MEDLINE, EMBASE (OVID), Cochrane Library, and PsychINFO to screen related studies prior to August 1, 2022. We included only studies that were reported in English. For identifying eligible studies in PubMed databases, the following search strategy was employed: ((excision repair cross-complementation group1) OR (ERCC1)) AND ((Cervical Neoplasm, Uterine) OR (Cervical Neoplasms, Uterine)) OR (Neoplasm, Uterine Cervical)) OR (Neoplasms, Uterine Cervical)) OR (Uterine Cervical Neoplasm)) OR (Neoplasms, Cervical)) OR (Cervical Neoplasms)) OR (Cervical Neoplasm)) OR (Neoplasm, Cervical)) OR (Neoplasms, Cervix)) OR (Cervix Neoplasms)) OR (Cervix Neoplasm)) OR (Neoplasm, Cervix)) OR (Cancer of the Uterine Cervix)) OR (Cancer of the Cervix)) OR (Cervical Cancer)) OR (Uterine Cervical Cancer)) OR (Cancer, Uterine Cervical)) OR (Cancers, Uterine Cervical)) OR (Cervical Cancer, Uterine)) OR (Cervical Cancers, Uterine)) OR (Uterine Cervical Cancers)) OR (Cancer of Cervix)) OR (Cervix Cancer))). The publication’s reference lists were manually checked to detect additional studies. On the basis of a data collection form, the following information was extracted, including the first authors’ names of the included studies, study publication year, the study type, median/mean age, stage of cervix cancer, treatment for cervix cancer, assessment for ERCC1 examination, the number of moderate/high/positive ERCC1 patients and low/negative ERCC1 patients, and the clinical implications or significances of ERCC1 in cervix cancer.

As shown in **Tables 1**–**3**, there were nineteen relevant studies (11, 12, 21–37) that were finally included for further evaluation. Among these eligible studies, thirteen studies were clinical trials reporting the ERCC1 expression and cervix cancer, three studies (33–35) were clinical studies reporting the ERCC1 polymorphism and cervix cancer, and three experimental studies (21, 36, 37) reporting the molecular roles of ERCC1 in cervix cancer. Study publication years ranged from 2000 to 2021 for the included studies. All the clinical studies were retrospective design. The median/mean age of the cervix cancer patients ranged from 43-58 years. The stage of cervix cancer patients included I to IVB, metastatic stage, recurrent stage, advanced stage, and locally advanced stage. The treatment methods for cervix cancer included radiation (i.e., EBRT), radical hysterectomy, neoadjuvant chemotherapy, chemoradiotherapy, and concurrent chemoradiotherapy. The common-used chemotherapeutic drugs among the included studies included etoposide, cisplatin, ifosfamide, fluorouracil (FU), cyclophosphamide (CTX), cyclophosphamide (CTP), etc. The assessments for evaluating the expression of ERCC1 mainly included immunohistochemistry (IHC), real-time polymerase chain reaction (RT-PCR), immunofluorescence, and fluorescence. The number of moderate/high/positive ERCC1 patients among the eligible clinical studies ranged from 7 to 72, while the number of low/negative ERCC1 patients in these studies ranged from 9 to 71.

**Table 1:** Clinical findings of ERCC1 in cervical cancer.

Study	Study type	Median/mean Age, Years	Stage	Treatment regimen	ERCC1 expression assessment	Moderate/high/positive ERCC1 patients (n)	Low/negative ERCC1 patients(n)	Clinical significances
Doll et al. (24)	Retrospectiv	NA	Locally advanced	Radiation	Fluorescent IHC	NA	NA	Patients with low ERCC1 expression had significantly worse OS (17.9% vs. 50.1%, P = 0.046) and worse DFS (21.4% vs. 47.4%, P= 0.083) than those with higher expression levels.
Hasegawa et al. (25)	Retrospectiv	46	FIGO Stage I to II	Radical hysterectomy	IHC	7	29	Patients with high ERCC1 expression had significantly worse DFS than those with low ERCC1 expression (P = 0.005). Similar trends were also observed in those patients received cisplatin-based chemotherapy or chemoradiotherapy with cisplatin (P=0.002).
Liang et al. (26)	Retrospectiv	54	Locally Advanced	Concurrent chemoradiotherapy	IHC	16	34	The 5-year disease-specific survival rates of the ERCC1-positive and ERCC1-negative groups were 43.8% vs. 76.5% (P = 0.011). The 5-year OS rates for the ERCC1-positive and ERCC1-negative groups were 50.0% vs. 85.3% (P = 0.008).
Park et al. (Park et al. (27))	Retrospectiv	50	Stage II B	Neoadjuvant chemotherapy (etoposide and cisplatin)	IHC	34	9	Response to chemotherapy was detected in all patients with negative ERCC1 expression. ERCC1 negativity was an independent predictor for responsiveness to neoadjuvant chemotherapy (P=0.021). Low ERCC1 expression was a significant prognostic factor of DFS in multivariate analysis (P=0.046).
Bai et al. (Bai et al. (28))	Retrospectiv	53	Locally Advanced	Chemoradiotherapy (cisplatin)	RT-PCR	29	31	Patients with low ERCC1 mRNA expression had a significantly higher rate of complete response (86.21%) than those with high level of ERCC1 (19.36%, P < 0.001).
Doll et al. (Doll et al. (29))	Retrospectiv	NA	Locally Advanced	Chemoradiation	Immunofluorescent	NA	NA	Tumoral ERCC1 status (nuclear to cytoplasmic ratio) was correlated to OS (HR=3.13, 95%CI: 1.27-7.71, P=0.013) and PFS (HR=2.33, 95%CI: 1.05-5.18, P=0.038).
Bajpai et al. (22))	Retrospectiv	43	NA	Chemoradiotherapy (cisplatin)	RT-PCR, Western blot	11	39	ERCC1 expressions were statistically lower in cervical cancer tissues than that in the normal cervix tissues (P=0.025)
Muallem et al (23))	Retrospective	44	advanced	EBRT and Cisplatin	IHC	72	40	The 2-year OS in the low, intermediate, and high ERCC1 group was 68.6%, 71.7%, and 90.7%, respectively. The 2-year PFS in the low, intermediate, and high ERCC1 group was 49.7%, 33.5%, and 72.7%, respectively.
KATO et al. (11)	Retrospectiv	46	Stage I B1-IV B	Nedaplatin	IHC	26	19	There were no significant differences in ERCC1 expression between the low and high sensitivity to nedaplatin groups (P=0.079).
Zwenger et al (30)	Prospective	43.5	advanced	Cisplatin	IHC	35	53	Poor DFS (P=0.021) and OS (P=0.005) were observed in cisplatin chemoradiotherapy patents with high ERCC1 expression.
Karageorgopoulou et al. (12))	Retrospective	58	metastatic/recurrent	Cisplatin and ifosfamide	IHC	32	11	Higher ERCC1 expression had shorter PFS and OS than those with low ERCC1 expression (mPFS: 5.1 vs 10.2 months, P = 0.027; mOS: 10.5 vs. 21.4 months, P = 0.006).
Ryu et al. (31)	Retrospective	51	IVB/metastatic/recurrent	Cisplatin	IHC	13	19	The median OS of ERCC1-high patients was 320 days and that of ERCC1-low patients was 617 days (HR=2.322, 95%CI: 1.051–5.129; P=0.037). The median PFS was significantly poorer in ERCC1-high than in ERCC1-low patients (135 vs 242 days; HR=2.428, 95%CI: 1.145–5.148; P=0.032).
Jeong et al. (32)	Retrospectiv	46	I B1 to II B	Chemoradioresistance	IHC	60	71	High ERCC1 expression suggested significantly unfavorable DFS (76.8% vs. 88.6%, P=0.022).

ERCC1, excision repair cross-complementation group1; IHC, immunohistochemistry; OS, overall survival; PD, progressive disease; HR, hazard ratio; PFS, progression free survival; DFS, disease-free survival; RT-PCR, real-time polymerase chain reaction.

**Table 2:** ERCC1 polymorphism in cervical cancer.

Study/Reference	Sample size	Examination sample/tissue and method	ERCC1 polymorphism	Main findings
HAN et al. (33)	Invasive cervical cancer: 229; non-cancer controls: 204	Peripheral blood; PCR restriction fragment length polymorphism assay	C19007T	The allelic frequencies of cancer patients were not significantly different from that of controls (P = 0.925); The C/C genotype had no increased risk for cervical cancer susceptibility compared with the TT genotype (P = 0.932). There was no significant relationship between the ERCC1 C19007T polymorphism and cervical cancer invasiveness (all P<0.05).
Zhang et al. (34)	Cervical cancer: 154; non-cancer controls: 177	Peripheral blood; SNPware 12plex assay	118C>T	ERCC1 118C>T was associated with high risk of cervical squamous cell carcinomas under additive genetic model and the dominant genetic model (all P< 0.05)
Soares et al. (35)	260 patents with cervical cancer who underwent cisplatin treatment	White blood cell; Allelic discrimination RT-PCR	rs3212986	An association between ERCC1 rs3212986 and the onset of late gastrointestinal toxicity underwent cisplatin treatment (P=0.038); Patients carrying AA homozygous genotype have an increased risk of developing late gastrointestinal toxicity as compared to patents with the C allele (OR = 3.727, 95%CI: 1.199-11.588, P= 0.017).

ERCC1, excision repair cross-complementation group1; OR, odds ratio; CI, confidence interval; RT-PCR, real-time polymerase chain reaction.

**Table 3:** Molecular mechanisms underlying the effects of ERCC1 in cervical cancer.

Study/Reference	Treatments for cervical cancer	Experimental model	Main findings
Britten et al. (36)	Cisplatin resistance	Cervical carcinoma lines (HT137, HT155, HT172, HT180 and HT212)	There was a significant correlation between ERCC1 mRNA expression and cisplatin resistance in all cervical carcinoma lines (all P< 0.05), but such an association was not significant in ERCC1 protein expression (all P>0.05). It might be possible to identify cervical tumors likely to be resistant to cisplatin by examining pre-treatment ERCC1 mRNA levels.
Torii et al. (21)	Cisplatin and 5-FU	Uterine cervical adenocarcinoma cells (HCA-1 and TCO-2)	There was an association between ERCC1 expression and sensitivity to cisplatin in cervical adenocarcinoma cells. A cisplatin-resistant cell line HCA-1R showed a dramatically higher level of ERCC1 mRNA expression than the native cells. Co-administration of cisplatin and 5-FU showed the synergistic or additive effects *via* inhibiting of ERCC1 expression.
Almeida et al. (37)	Radiotherapy	CASKI and C33A cells	Absent or weak modulations of ERCC1 was detected after exposure to 1.8 Gy of radiotherapy in cell lines, which might be associated with the inhibition of the regulatory axis p53-EGFR-ERCC1. Increased expressions of ERCC1 (5/10 patients; P=0.0294) was found in malignant tissues after radiotherapy with the same radiation dose. This study showed that upregulation of ERCC1 may be part of a radioresistance mechanism in cervical cancer.

ERCC1, excision repair cross-complementation group1; EGFR, epidermal growth factor receptor.

In the three clinical studies reporting the ERCC1 polymorphism, the sample size ranged from 260 to 433. The results of polymorphism examination derived from the peripheral blood and white blood cell. The methods for polymorphism detection in these studies included PCR restriction fragment length polymorphism assay, SNPware 12plex assay, and allelic discrimination RT-PCR. The reported ERCC1 polymorphisms among the three studies were C19007T, 118C>T, and rs3212986.

There were three experimental studies that investigated the aberrant expression of ERCC1 in cervix cancer. The research models among these studies were all *in-vitro* designed, which included a variety of cervical carcinoma lines, i.e., HT137, HT155, HT172, HT180, HT212, CASKI, and C33A cells. These cancer cells were treated with cisplatin resistance, 5-FU, and radiotherapy. A summary of the nineteen studies included in this study can be found in **Tables 1**–**3**.

### Pro-oncogenic effects of ERCC1 in FIGO stage I to Stage III uterine cervix cancer

Currently, there is evidence that ERCC1 contributes to resistance to platinum-based chemotherapy or chemoradiotherapy coupled with platinum agents in multiple malignancies (38). For example, the relationship between ERCC1 expression and clinical characteristics and outcomes in patients with uterine cervical cancer has been detected in a number of studies. Such an association was not only observed in the early stage but also the advanced stage of uterine cervix cancer. According to the published data, high expression of ERCC1 might be correlated with poor prognosis in cervix cancer. Hasegawa et al. (25) reported that patients with FIGO stage I to II uterine cervix cancer with high ERCC1 expression had significantly worse DFS than those with low ERCC1 expression (P = 0.005). In addition, worse DFS was also observed in those patients who had a high level of ERCC1 under cisplatin-based chemotherapy/chemoradiotherapy (*P*= 0.002). The log-rank test indicated that high ERCC1 expression might be an independent prognostic factor in patients receiving cisplatin treatment (P<0.05). This finding was consistent with Park et al.’s study (27) which investigated the roles of ERCC1 in patients with Stage II B cervix cancer under neoadjuvant chemotherapy (etoposide and cisplatin). It was found that chemotherapy was responsive in all patients with negative ERCC1 expression. ERCC1 negativity was an independent predictor for responsiveness to neoadjuvant chemotherapy (*P=*0.021). This study also reported that low ERCC1 expression was a significant prognostic factor of DFS in multivariate analysis (*P=*0.046). In a more recent study (32) developed by Jeong et al., the authors investigated the prognostic significance of ERCC1 in early-stage (FIGO I B1 to II B) cervical cancer with chemoradioresistance. They observed that high ERCC1 expression was associated with significantly unfavorable DFS than those with low ERCC1 expression (76.8% vs. 88.6%, *P=*0.022). The above three clinical studies demonstrated that ERCC1 might play a pro-cancer role in early-stage uterine cervix cancer, especially in patients with cisplatin chemotherapy.

### Pro-oncogenic Effects of ERCC1 in advanced uterine cervix cancer

In addition to the early stage of uterine cervix cancer, ERCC1 expression was also found to be associated with the prognosis of advanced cervix adenocarcinoma. An early study conducted by Bai et al. (28) demonstrated that advanced cervical squamous cell carcinoma patients with low ERCC1 mRNA expression had a significantly higher rate of complete response to cisplatin-based concurrent chemoradiotherapy (86.21%) than those with a high level of ERCC1 (19.36%, P < 0.001). Further analysis indicated that low ERCC1 mRNA level was an independent predictive factor for a complete response to chemoradiotherapy (P < 0.001). The authors also found that the sensitivity for detecting a complete response was 81.48% with a specificity of 96.97%. Liang et al. (26) investigated the clinical outcome in patients administrated with cisplatin-based concurrent chemoradiotherapy for locally advanced cervical cancer. They found that the 5-year DFS rates of the ERCC1-positive and ERCC1-negative groups were 43.8% vs. 76.5% (P = 0.011) and the 5-year OS rates for the ERCC1-positive and ERCC1-negative groups were 50.0% vs. 85.3% (P = 0.008). Zwenger et al. (30) demonstrated that poor DFS (*P=*0.021) and OS (*P=*0.005) were observed in patients with advanced cervical cancer who received cisplatin chemoradiotherapy with high ERCC1 expression when compared to those with low ERCC1 levels.

In addition to the above evidence, A correlation was also found between ERCC1 expression and survival in patients with metastatic or recurrent uterine cervix carcinoma treated with cisplatin and ifosfamide. Karageorgopoulou et al. (12) demonstrated that higher ERCC1 expression had shorter PFS and OS than those with low ERCC1 expression (median PFS: 5.1 vs 10.2 months, P = 0.027; median OS: 10.5 vs. 21.4 months, P = 0.006). Similarly, a study done in Korea showed the median OS of ERCC1-high patients was 320 days and that of ERCC1-low patients was 617 days (HR=2.322, 95%CI: 1.051–5.129; *P=*0.037) *(* 31). Also, the median PFS was significantly poorer in ERCC1-high than in ERCC1-low patients (135 vs 242 days; HR=2.428, 95%CI: 1.145–5.148; *P=*0.032) *(* 31). These preliminary studies indicated the prognosis and survival of patients with metastatic and recurrent uterine cervix cancer is poor when high ERCC1 expression is confirmed.

**Figure 1:**
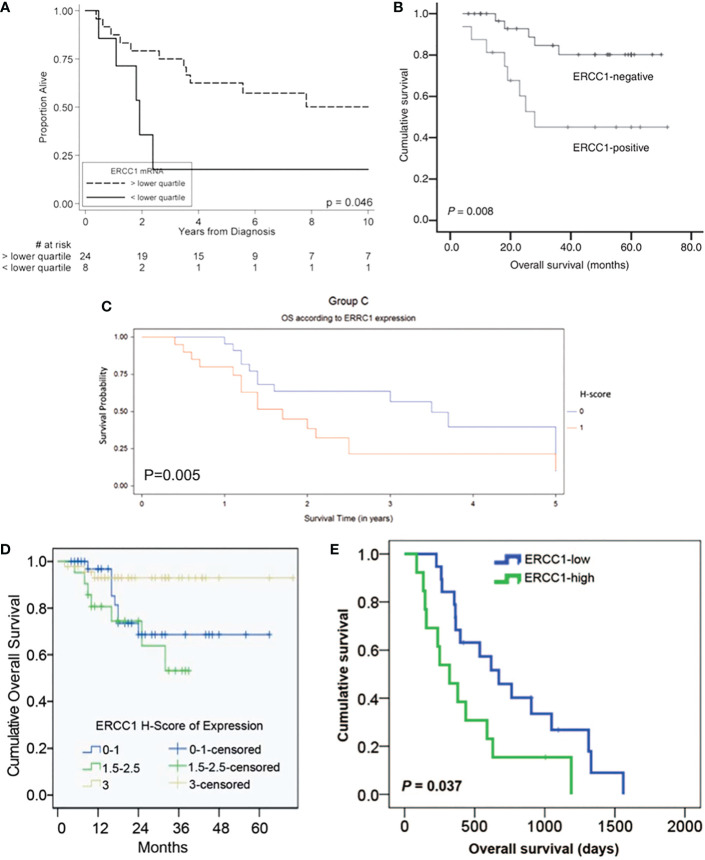
Kaplan-Meier overall survival curve stratified by ERCC1 status that reported in five included studies with the corresponding citation. **(A)** derived from the study of (24), namely **A** = (24); **B** = (29); **C** = (30); **D** = (23); **E** = (31).

**Figure 2:**
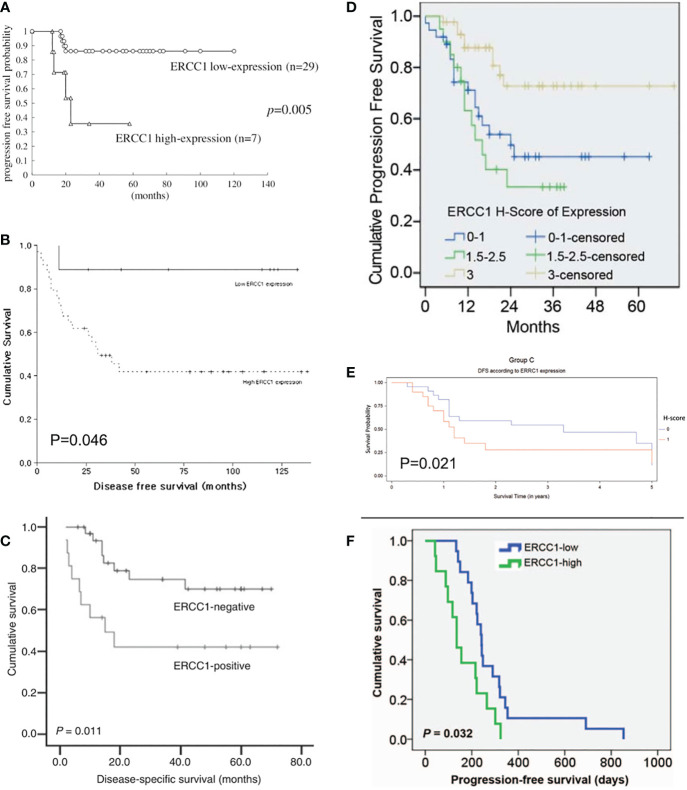
Kaplan-Meier disease-free and progression-free survival curve stratified by ERCC1 status that reported in six included studies with the corresponding citation. **(A)** derived from the study of (25), namely **A** = (25); **B** = (26); **C** = (29); **D** = (23); **E** = (30); **F** = (31).

The Kaplan-Meier OS, PFS, and DFS curves stratified by ERCC1 status that reported in the included studies were displayed in **Figures 1**, **2**.

### ERCC1 serves as a tumor suppressor in advanced uterine cervix cancer

Inconsistencies from the above studies, Bajpai et al. (22) indicated that the level of ERCC1 was statistically lower in cervical cancer tissues than that in the normal cervix tissues (*P=*0.025) in patients under chemoradiotherapy (cisplatin combined with radiotherapy). Doll et al. (24) reported that uterine cervix cancer patients with low ERCC1 expression had significantly worse OS (17.9% vs. 50.1%, *P* = 0.046) and worse DFS (21.4% vs. 47.4%, *P=* 0.083) than those with higher expression levels. Also, in a subsequent study developed by Doll et al. (29), they observed that tumoral ERCC1 status (nuclear to cytoplasmic ratio) was dramatically associated with the OS of the patients with cervical cancer (HR=3.13, 95%CI: 1.27-7.71, *P=*0.013) as well as correlated with the PFS (HR=2.33, 95%CI: 1.05-5.18, *P=*0.038). Based on the results from Doll et al., patients with cervical cancer who expressed high levels of ERCC1 were thought to have a better survival.

Consistent with Doll et al.’s findings, Muallem et al. (23) also indicated that the high level of ERCC1 was associated with poor prognosis for patients with malignant cervical carcinoma and this tendency was presented as a “dose-response”. It was reported that the 2-year OS of advanced cervical cancer patients in the low, intermediate, and high ERCC1 group was 68.6%, 71.7%, and 90.7%, respectively (23). However, such trend in PFS was not always the same as the tendency of OS. It was reported that the 2-year PFS in the low, intermediate, and high ERCC1 group was 49.7%, 33.5%, and 72.7%, respectively (23). Overall, these results showed thatpatients with advanced cervical cancer who have a low level of ERCC1 have a worse OS and PFS.

Of note, some studies have also shown that ERCC1 expression does not have a clinical significance in patients with cervical cancer. For example, a previous trial conducted in Japan had recruited 45 patients with Stage I B1-IV B carcinoma of the cervix and found that there were no significant differences in ERCC1 expression between the low and high sensitivity to nedaplatin groups (*P=*0.079) (11). As a result of this study, it was suggested that ERCC1 was not an essential component of the cervical cancer process.

### ERCC1 polymorphism and the risk of cervical cancer in women

Genetic mutagenesis can be caused by DNA alterations under environmental or endogenous carcinogens, leading to carcinogenesis (39). Single nucleotide polymorphisms (SNPs) are proposed to be one of the important biomarkers in the prognosis and therapeutic response of oncologic patients (40). In this comprehensive review, there were three studies (**Table 2**) reporting the association between ERCC1 polymorphisms and the risk of cervical cancer. Zhang et al. (34) analyzed the ERCC1 polymorphisms in peripheral blood from 154 cervical cancer patients and 177 non-cancer controls. The results showed that ERCC1 118C>T was associated with a high risk of cervical squamous cell carcinomas under the additive genetic model and the dominant genetic model (all P< 0.05). Platinum agents and ionizing radiation can induce hematological toxicities, genitourinary toxicity, and gastrointestinal toxicity (41). In a more recent study, Soares et al. (35) demonstrated that there was an association between ERCC1 rs3212986 and the onset of late gastrointestinal toxicity underwent cisplatin treatment (*P=*0.038). Patients carrying AA homozygous genotype had an increased risk of developing late gastrointestinal toxicity as compared to patients with the C allele (OR = 3.727, 95%CI: 1.199-11.588, *P=* 0.017). The underlying mechanisms might be correlated to the altered DNA repair capacity induced by ERCC1 rs3212986 polymorphism. However, some researchers in Korea did not find a positive association between ERCC1 polymorphisms and cervical cancer by evaluating the peripheral blood through the PCR restriction fragment length polymorphism assay in 229 invasive cervical cancer patients and 204 non-cancer controls (33). The allelic frequencies of ERCC1 in cervical cancer patients were not significantly different from those of the controls in this study (P = 0.925). The C/C genotype had no increased risk for cervical cancer susceptibility compared with the TT genotype (*P* = 0.932) (33). The authors concluded that there was no significant relationship between the ERCC1 C19007T polymorphism and cervical cancer invasiveness in Korean women (all *P*<0.05) *(* 33).

Based on the above 3 included studies, 67% (2/3) of them suggested there was a positive relationship lying in ERCC1 polymorphism and the development and therapeutic response of cervical cancer. Since the genetic polymorphisms often vary between ethnic groups, the clinical outcomes of ERCC1 polymorphism might be not significant. Even though, detection of ERCC1 polymorphism might be a useful method for implementing strategies when choosing a proper treatment for a patient so as to reduce the toxicities or improve the treatment response rates in cervical cancer women.

### Roles of ERCC1 in cervical cancer reported in experimental studies

Three *in-vitro* studies (**Table 3**) reported the molecular mechanisms of ERCC1 in cervical cancer that were available in the literatures. Cisplatin is one of the valuable adjuvants to radiotherapy for treating cervical cancer (42). However, patients are at risk for developing drug-resistant cervical cancer due to the progression of the disease. Britten et al. (36) developed several cervical carcinoma cell lines (e.g. HT137, HT155, HT172, HT180, and HT212) of cisplatin resistance. The authors found that there was a significant correlation between ERCC1 mRNA expression and cisplatin resistance in all cervical carcinoma lines (all P< 0.05), but such an association was not significant in ERCC1 protein expression (all P>0.05) (36). According to this study, it might be possible to identify cervical tumors likely to be resistant to cisplatin by examining pre-treatment ERCC1 mRNA levels.

It was suggested that combined chemotherapy had additive or synergistic effects on various specific malignancies, which could significantly prolong the survival of the sufferers (43). Torii et al. (21) examined the expression of ERCC1 in uterine cervical adenocarcinoma cells treated with cisplatin and 5-FU. The results turned out that a positive association between ERCC1 expression and sensitivity to cisplatin in cervical adenocarcinoma cells (HCA-1 and TCO-2). Cancer cells treated with cisplatin resulted in a significant elevation of ERCC1 expression, while a cisplatin-resistant cell line HCA-1R presented with a dramatically higher level of ERCC1 mRNA expression than the native cells. Interestingly, co-administration of cisplatin and 5-FU remarkably reduced the expression of ERCC1 in both HCA-1 and HCA-1R cells. Thus, co-administration of cisplatin and 5-FU showed synergistic or additive effects *via* inhibiting of ERCC1 expression, indicating a clinical advantage of combining these two drugs for suppressing ERCC1 in cervical adenocarcinoma cells. From the point of view of ERCC1 suppression, such combination therapy with cisplatin and 5-FU might be a promising treatment regimen for cervical adenocarcinoma.

Cisplatin-based chemotherapy and radiotherapy are the common-used combined treatments for locally advanced cancer diseases, while radiotherapy alone is considered to be applied for patients with early disease (44). Almeida et al. (37) conducted a clinical and experimental study. Immunohistochemical analysis on the tissues of the patients showed that increased expressions of ERCC1 (5/10 patients; *P=*0.0294) were found in malignant tissues after radiotherapy. An elevated expression of ERCC1 was found in half of the patients after treatment with 1.8 Gy. *In-vitro* experiments suggested that absent or weak modulations of ERCC1 were detected after exposure to 1.8 Gy of radiotherapy in cervical cell lines. The authors also supposed that the mechanisms might be correlated with the inhibition of the regulatory axis p53-EGFR-ERCC1 in tumor cells exposed to radiation *in vivo* (37). This study showed that the upregulation of ERCC1 might be part of a radio-resistance mechanism in cervical cancer.

### Other molecular mechanisms underlying ERCC1 expression and cervical cancer

ERCC1 is one of the DNA repair genes (45). Its enzyme involves the nucleotide excision repair pathway that recognizes and eliminates cisplatin-associated DNA adducts (13, 46). One proposed mechanism for ERCC1 in cancer development might be due to the aberrant expression of ERCC1 causing the dysfunction of DNA-repair capacity, leading to the accumulation of genetic damage, which might induce the emergence of an aggressive tumor phenotype (47). ERCC1 status represents both the cellular intrinsic DNA damage repair ability and the extent of accumulated intratumoral DNA damage, which may be associated with the progression of the cancers (48). Besides, abnormal ERCC1 expression resulted in genetic instability and thus affected the therapeutic response under cisplatin to radiotherapy. Human gliomas seem to be resistant to cisplatin because of hypermethylation of the promoter of the ERCC1 gene (49).

Affected genes and signaling pathways might contribute to the effects of ERCC1 in cervical cancer. The 3’ side incision by ERCC4 requires ERCC1, which is located on chromosome 19. The ERCC1-ERCC4 complex was found to play roles in interstrand cross-link repair induced by the recombination repair mechanisms (22). ERCC1 is an endonuclease, serving as a heterodimer with xeroderma protein F (XPF). ERCC1/XPF complexes play roles in the incision that cleaves the damaged nucleotide strand at the 5’ end of the lesion (50). ERCC1 exerts effects on the response to a range of DNA-damaging chemotherapeutic agents. It was reported that ERCC1 might act together with class III β-tubulin (TUBB3), which was jointly involved in the development of locally advanced cervical squamous cell carcinoma (30).

**Figure 3:**
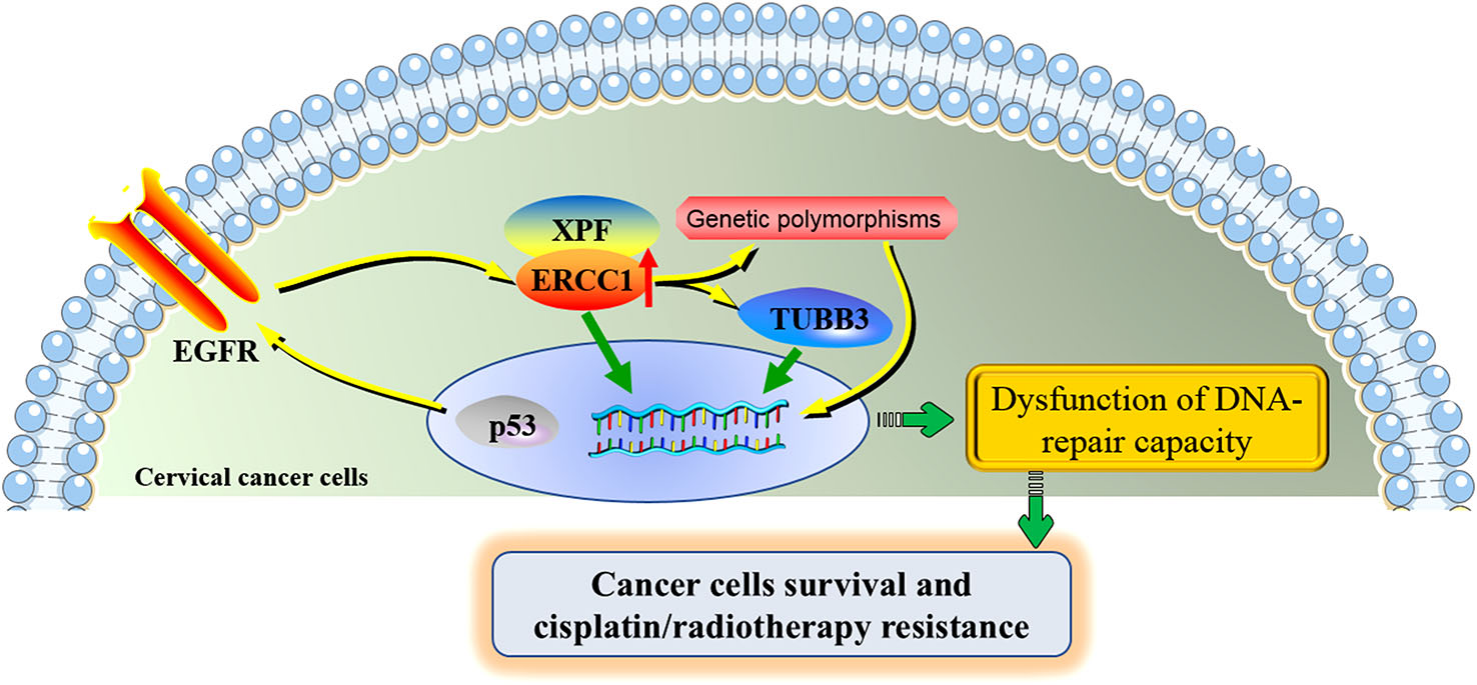
The potential molecular mechanisms underlying the roles of ERCC1 in cervical cancer. ERCC1 involves in the development and cisplatin/radiotherapy resistance in cervical cancer through the interaction with several specific genes and genetic polymorphisms. ERCC1 and XPF form a heterodimeric protein complex that cleaves the damaged nucleotide strand at the 5’ end of the lesion. ERCC1 acts together with TUBB3, contributing to the poor prognosis of cervical cancer. The activation of the regulatory axis p53-EGFR-ERCC1 may be part of a radio-resistance mechanism in cervical cancer. ERCC1 genetic polymorphisms partially contribute to the progression of cervical cancer and the toxicities under cisplatin treatment. Aberrant expression of ERCC1 and its associated genes and affected signaling pathways were jointly involved in the dysfunction of DNA-repair capacity of cervical cancer cells, increasing the proliferation of cervical cancer cells and allowing the resistance of cisplatin/radiotherapy. ERCC1, excision repair cross-complementation group 1; XPF, xeroderma pigmentosum group F; TUBB3, class III β-tubulin; EGFR, epidermal growth factor receptor.

The potential molecular mechanisms underlying the roles of ERCC1 in cervical cancer were shown in **Figure 3**.

### Potential roles of targeting of ERCC1 in cervical cancer

As aforementioned, mounting clinical studies have confirmed the outstanding prognostic effects of ERCC1 in cervical cancer, thus the development of immunotherapy by targeting ERCC1 (i.e., ERCC1 inhibitor) may have important implications for modulating the antitumor immune responses in patients with advanced cervical cancer. There is a tight relationship between chemotherapy resistance and immunosuppression (51). In this review, ERCC1 expression was found to be correlated to chemotherapy-resistance (i.e., cisplatin and 5-FU) in cervical carcinoma, chemotherapy combined with ERCC1 inhibitor may dramatically reduce the immunosuppression and thus reinstate the immune function.

ERCC1 inhibitor may be not only applied for the combination with chemo/radiotherapy, but also the immunotherapy with check point inhibitors (i.e., anti-PD1 and anti-CTLA4). Combination of anti-PD-1 plus anti-CTLA-4 immunotherapy shows greater response rates than anti-PD-1 or anti-CTLA-4 antibody alone in multiple malignancies (52, 53). Due to a different anti-tumor mechanism of antitumor agents in a specific cancer type, a combination of drugs is recommended. For example, the combination of anti-PD-1 inhibitor and bevacizumab (an anti-vascular endothelial growth factor (VEGF) antibody, namely VEGF inhibitor) was found to have better outcomes in patients compared to sorafenib (54). Poly(ADP-ribose) polymerase inhibitor (PARPi) exerts therapeutic effect on various types of cancers. Trapping of PARP on the DNA by a small molecule PARPi generates DNA-PARP complexes. The capability of DNA repair is subsequently suppressed, resulting in replication fork collapse and catastrophic DNA double strand breaks which are selectively lethal to the cancer cell (55). It was reported that targeting PARP-1 with metronomic therapy might enhance anti-PD-1 immunotherapy in colon cancer (56). Similarly, since ERCC1 serving as a key DNA repair gene, ERCC1 inhibitor may be also applied for combining immunotherapy with check point inhibitors, which may help to enhance antitumor efficacy. Thus, ERCC1 inhibitor combined with either traditional regimens (i.e., chemotherapy or radiotherapy) or lately immunotherapies (i.e., anti-PD1, anti-CTLA4, or both) may obtain promising antitumor efficacy on cervical cancer.

### Directions for future research

Cervical cancer is a public health problem of extensive clinical importance (57). Based on the above evidence from both clinical and experimental studies, ERCC1 is one of the essential and important factors in the progression of carcinoma of the uterine cervix and the therapeutic response of chemoradiotherapy. However, there are several points worth noting when interpreting the results. First, in this review, the relationship between ERCC1 expression and the status of cisplatin-based treatments in early and advanced cervical cancer has been extensively studied. However, the association between ERCC1 expression and chemosensitivity to other common chemotherapeutic medicines has not been fully investigated. Second, ERCC1 polymorphisms might also play roles in predicting the risk of cervical cancer and the toxicities that underwent cisplatin treatment, but whether these polymorphisms function in patients’ survival has not been elucidated. Third, the exact and in-depth molecular mechanisms underlying the effects of ERCC1 expression and the development of cervical cancer are not clear due to limited studies and need to be further elucidated. Therefore, more clinical, *in-vitro*, and *in-vivo* investigations are still warranted for future studies. Fourth, the importance of the development of immunotherapy trials by targeting ERCC1, i.e., ERCC1 inhibitor, should be addressed in the future.

## Conclusion

The present review highlights the crucial roles of ERCC1 expression in cervical cancer. The majority of the included studies suggested that the ERCC1 served as a pro-oncogenic factor in both early-stage and advanced cervix cancer due to high expression of ERCC1 has been found to be associated with poor survival of the patients. ERCC1 polymorphism detection might be a useful tool for predicting the risk of cervical cancer and the toxicities that underwent cisplatin treatment. Experimental studies suggested that the biological effects exerted by ERCC1 in cervical cancer might be mediated by its associated genes and affected signaling pathways. To move toward clinical applications by targeting ERCC1 in cervical cancer, more investigations are still warranted in the future.

## Author contributions

PD and GL contributed to the design of the study. LW and MH conducted the systematical search and extracted the clinical and experimental data. PD and GL wrote the manuscript. LW and MH supervised the manuscript. All authors contributed to the article and approved the submitted version.
